# Peripheral neuroectodermal tumor in the nasal cavity – a case report^☆^

**DOI:** 10.1016/j.bjorl.2017.05.006

**Published:** 2017-06-02

**Authors:** Daniel Marcus San da Silva, Ramon Nobre Leal Oliva, Vitor Guo Chen, Maria Teresa de Seixas Alves, Reginaldo Raimundo Fujita

**Affiliations:** a Universidade Federal de São Paulo (UNIFESP), São Paulo, SP, Brazil; b Universidade Federal de São Paulo (UNIFESP), Departamento de Otorrinolaringologia e Cirurgia de Cabeça e Pescoço, Disciplina de Otorrinolaringologia Pediátrica, São Paulo, SP, Brazil; c Universidade Federal de São Paulo (UNIFESP), Departamento de Patologia, São Paulo, SP, Brazil

## Introduction

Primitive neuroectodermal tumors (PNETs) comprise a group of tumors originated from the neuroectoderm-ectoderm and are related to tumors classified as Ewing's sarcoma. When they occur outside the central nervous system, they are called peripheral (pPNET). They occur mainly in the chest and extremities and the involvement of head and neck structures is rare, especially in children. Among these structures, the most commonly affected site is the orbit, followed by the neck and parotid glands.^1^ There are no reports in the literature of exclusive nasal fossa lesions, and the tumors found in this site primary affect the maxillary sinus.^2^ They are extremely aggressive tumors, and carry a poor prognosis. The aim of this report is to describe the initial presentation and evolution of this tumor in the nasal cavity in a male adolescent, first seen in the Otorhinolaryngology Emergency Room (ORL/ER), and later referred to the pediatric oncology referral service.

## Case report

**Figure 1:**
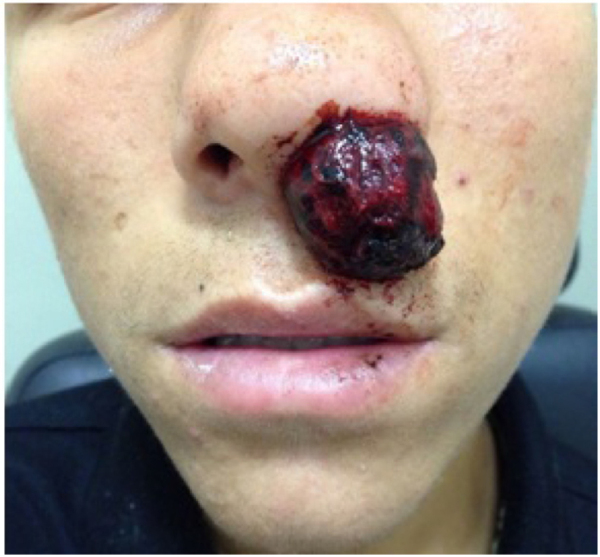
Tumor lesion of the left nasal cavity.

A 15-year-old male patient, previously healthy and without comorbidities, reported self-limited episodes of epistaxis in the left nasal cavity since January 2015. He started experiencing progressive nasal obstruction with tumor exteriorization in June 2015, when he sought the ORL/ER (Fig. 1). The tumor was friable and had an irregular surface, protruding from the left nostril, was of purplish color and associated with diffuse bleeding in small amounts. There were no alterations in oroscopy and otoscopy.

**Figure 2:**
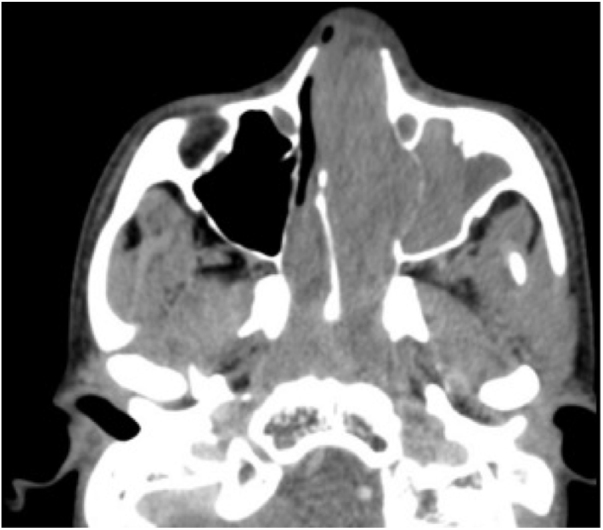
Facial sinus tomography.

**Figure 3:**
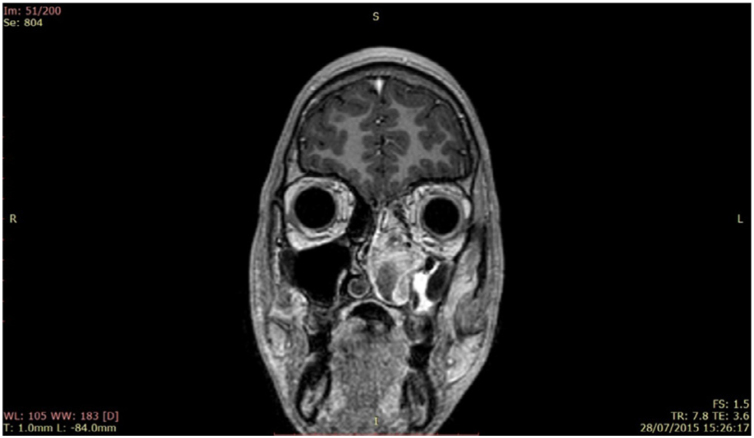
Lesion in the left nasal cavity.

The patient was referred to the Pediatric Otorhinolaryngology Outpatient Clinic after undergoing laboratory tests and computed tomography of the paranasal sinuses, which showed an expanding heterogeneous formation with poorly defined limits, occupying the rhinopharynx, maxillary, sphenoidal, and ethmoidal sinuses on the left, middle and lower meatuses, as well as the left nasal cavity (Fig. 2). Magnetic resonance imaging of the face with contrast showed that the lesion did not invade the orbit or the base of the skull (Fig. 3). On the day before the consultation, spontaneous detachment of the external part of the tumor occurred without major bleeding and on the day of the consultation, the patient brought the detached material, which was sent for anatomopathological analysis.

Analysis of the lesion disclosed a primitive neuroectodermal tumor, and immunohistochemistry was positive for markers of neuroendocrine tumors. He underwent biopsy and immunohistochemistry (IHC) analysis of the bone marrow, with negative results for neoplasia.

After diagnostic confirmation, the patient was referred to the pediatric oncology service, where investigation showed no metastatic lesions. The chosen treatment consisted of chemotherapy (Ewing Protocol) and radiotherapy with 25 sessions of 180 cGy in the tumor bed pre-chemotherapy and boost with 31 sessions 180 cGy in the tumor bed post-chemotherapy. The service to which the patient was referred chose not to perform surgery due to greater experience with radiotherapy and chemotherapy. Since the beginning of treatment, the patient has shown poor adherence to follow-up and clinical treatment. Currently, he has continued with radiotherapy after the completion of the chemotherapy, with the patient missing several of the consultations. There was significant regression of the tumor without worsening of the overall clinical status or evidence of metastases. Epistaxis episodes ceased and there was a gradual improvement in left nasal cavity obstruction.

## Discussion

The pPNETs belong to the Ewing's sarcoma family, comprising neoplasms of primitive neuroectodermal cells, which are embryonic cells that migrate from the neural crest. They are classified according to neural differentiation, with Ewing's sarcoma being an undifferentiated tumor, whereas pPNETs are tumors with neural differentiation.^3^

Nasal cavity tumors comprise 35% of sinonasal tumors, with squamous cell carcinoma accounting for 70–80% of cases.^4^ Head and neck pPNETs are extremely rare, and when found in the area, they are common in the orbital region, followed by the neck and parotid glands, respectively. The most common locations involve the thoracopulmonary region (called Askin's tumor when it affects the chest wall), abdomen, and extremities.^1^ They have a higher incidence in young individuals.^3^

Differential diagnosis includes nasal cavity pathologies that may develop into exophytic lesions, such as granulomatous diseases (reactions to foreign bodies, inflammatory, fungal, parasitic, autoimmune diseases), benign tumors of epithelial origin (inverted papilloma, cylindrical papilloma, keratotic adenoma), benign tumors of non-epithelial origin (osteoma, fibroma, chondroma, hemangioma, neurofibroma and neurilemmoma) and squamous cell carcinoma.^5^, ^6^ Considering the epidemiology shown by the patient, a diagnosis of juvenile nasoangiofibroma was suspected at the beginning of the investigation. Although squamous cell carcinoma is the main tumor of the nasal fossa, its incidence only becomes significant after the fifth decade of life.

**Figure 4:**
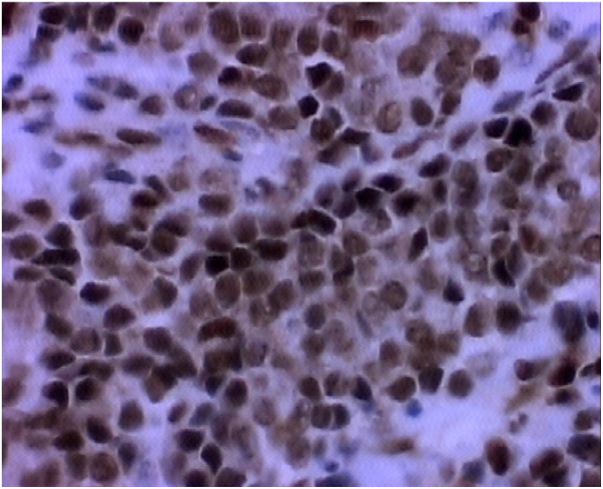
Photomicrography (400×) IHC FLI-1.

**Figure 5:**
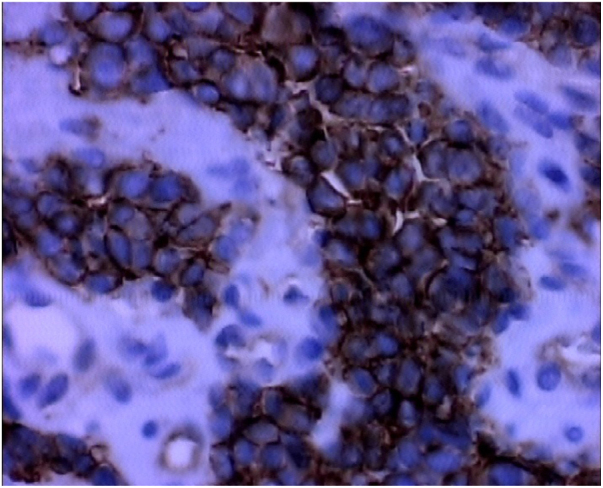
Photomicrography (400×) IHC CD99.

The investigation is initiated by the clinical and imaging examination, and the diagnosis is confirmed by histology and IHC. The most common symptom is nasal obstruction, but pain, epistaxis and visual alterations may occur.^7^ Computed tomography and magnetic resonance imaging are the examinations of choice. Histologically, PNET demonstrates small, rounded and poorly-differentiated cells. IHC has positive neuroendocrine tumor markers, and in our case, they were positive for enolase, synaptophysin, MIC 2 (CD99), FLI-1, vimentin and CD 34 positive for vessels (Figure 4, Figure 5). MIC 2 has high sensitivity, but low specificity.^1^ Most tumors belonging to the Ewing sarcoma family exhibit the t(11;22) (q24; 2) translocation, responsible for the EWS/FLI-1 fusion gene, producer of anti-FLI-1 antibody present in 85–90% of PNETs.^1^

There are no treatment protocols yet for pPNETs, but the literature demonstrates early tumor resection and multiple chemotherapy sessions as an option to treat residual disease, metastasis, or recurrence.^2^ Radiotherapy is chosen for patients without surgical management or with inadequate tumor resection.^2^ In our case we chose chemotherapy and radiotherapy. The patient is undergoing radiotherapy and shows poor adherence to the treatment; nevertheless, he remains asymptomatic, with stable disease.

The prognosis of pPNET is poor, with a survival of less than 50% in 3 years and 30–45% in 5 years.^1^ Factors such as tumor size, location, metastasis at disease onset, and initial response to chemotherapy are prognostic determinants. There is a tendency for the development of metastases to the lung, liver, and bone marrow.

## Conclusion

Although rare in head and neck topography, we cannot rule out the pPNET hypothesis in young patients with tumors in the nasal cavity, since it has a higher incidence in young individuals. The literature indicates that early tumor resection associated with chemotherapy and radiotherapy is the indicated type of treatment. In our case, chemotherapy and adjuvant radiotherapy were chosen due to the experience of the service where the patient was referred to, in which he is currently still being followed.

## Conflicts of interest

The authors declare no conflicts of interest.
